# Two vs. Three Incisions for Hypoglossal Nerve Stimulator Implantation to Treat Sleep Apnea: A Systematic Review

**DOI:** 10.7759/cureus.104514

**Published:** 2026-03-01

**Authors:** Amanda Kettman, Annah M Smelley, Samantha Main, Rahul Garg

**Affiliations:** 1 Research, Alabama College of Osteopathic Medicine, Dothan, USA

**Keywords:** a systematic review, hypoglossal nerve stimulator, obstructive sleep apnea (osa), satisfactory surgical outcomes, sleep quality and performance

## Abstract

Obstructive sleep apnea (OSA) is common among adults in the United States. Hypoglossal nerve stimulation (HNS) is a novel surgical alternative for OSA patients who are nonadherent to first-line treatments. Our systematic review evaluated the surgical and device outcomes of the traditional three-incision versus the newer, less-invasive two-incision HNS implantation technique. Four studies were included based on the inclusion and exclusion criteria. Two studies demonstrated a significant reduction in operative time from three-incision to two-incision (128.7 to 86.6 minutes and 143.3 to 129.4 minutes, respectively). Two studies reported better sleep waveform quality and non-inferior sleep quality index scores, and one study showed fewer follow-up corrective surgeries with the two-incision group (0% vs. 5.4%; p=0.048). Across most studies, postoperative complications were low. The sleep quality improved with both techniques without statistical significance. Based on limited evidence from observational studies, the two-incision approach shortened operative time and had fewer corrective follow-up surgeries without compromising sleep quality relative to the three-incision technique.

## Introduction and background

Obstructive sleep apnea (OSA) is characterized by recurrent episodes of upper airway obstruction during sleep, leading to intermittent sleep hypopnea or apnea ​[1].​ These episodes of airflow reductions, measured by the apnea-hypopnea index (AHI), disrupt normal sleep patterns and can lead to significant cardiovascular, metabolic, and neurocognitive complications ​[2,3,4]. Obstructive sleep apnea remains highly prevalent in the United States and is frequently undertreated due to intolerance or limited effectiveness of first-line therapies such as continuous positive airway pressure (CPAP) [5,6] and other oral appliances. The limitations of CPAP and other oral appliances have led to an increasing interest in surgical treatment options such as hypoglossal nerve stimulator (HNS) [7-12]. 

The HNS, approved in 2014, delivers electrical stimulation to the hypoglossal nerve to increase genioglossus tone and mitigate upper airway collapse, improving sleep-related outcomes in selected patients ​[13]. It achieves better compliance than a CPAP device, effectively reduces AHI and oxygen desaturation index, and improves patient-reported outcomes such as daytime sleepiness [13,14].​ Traditionally, HNS implantation has been performed using a three-incision approach, consisting of a submandibular incision for the placement of a stimulation lead, an anterior chest incision for the implantable pulse generator, and a lateral chest incision for the respiratory sensing lead [14]. In 2021, a less invasive two-incision technique allowed placement of the pulse generator and sensing lead via a single lateral chest incision while maintaining the second incision at the submandibular region. This modification aims to reduce operative time and surgical morbidity while maintaining therapeutic efficacy ​[14,15]. 

While technical considerations and clinical pathways for HNS implantation have been described previously, a focused synthesis comparing these two surgical incision techniques is lacking [16]. Though some retrospective studies have evaluated feasibility and short-term outcomes, a comprehensive comparative synthesis of patient outcomes related to the two techniques is needed. Our systematic review compared the surgical and device-related outcomes associated with two-incision and three-incision HNS implantation techniques in patients with OSA. 

## Review

Methods 

Study Design and Eligibility Criteria 

**Figure 1:**
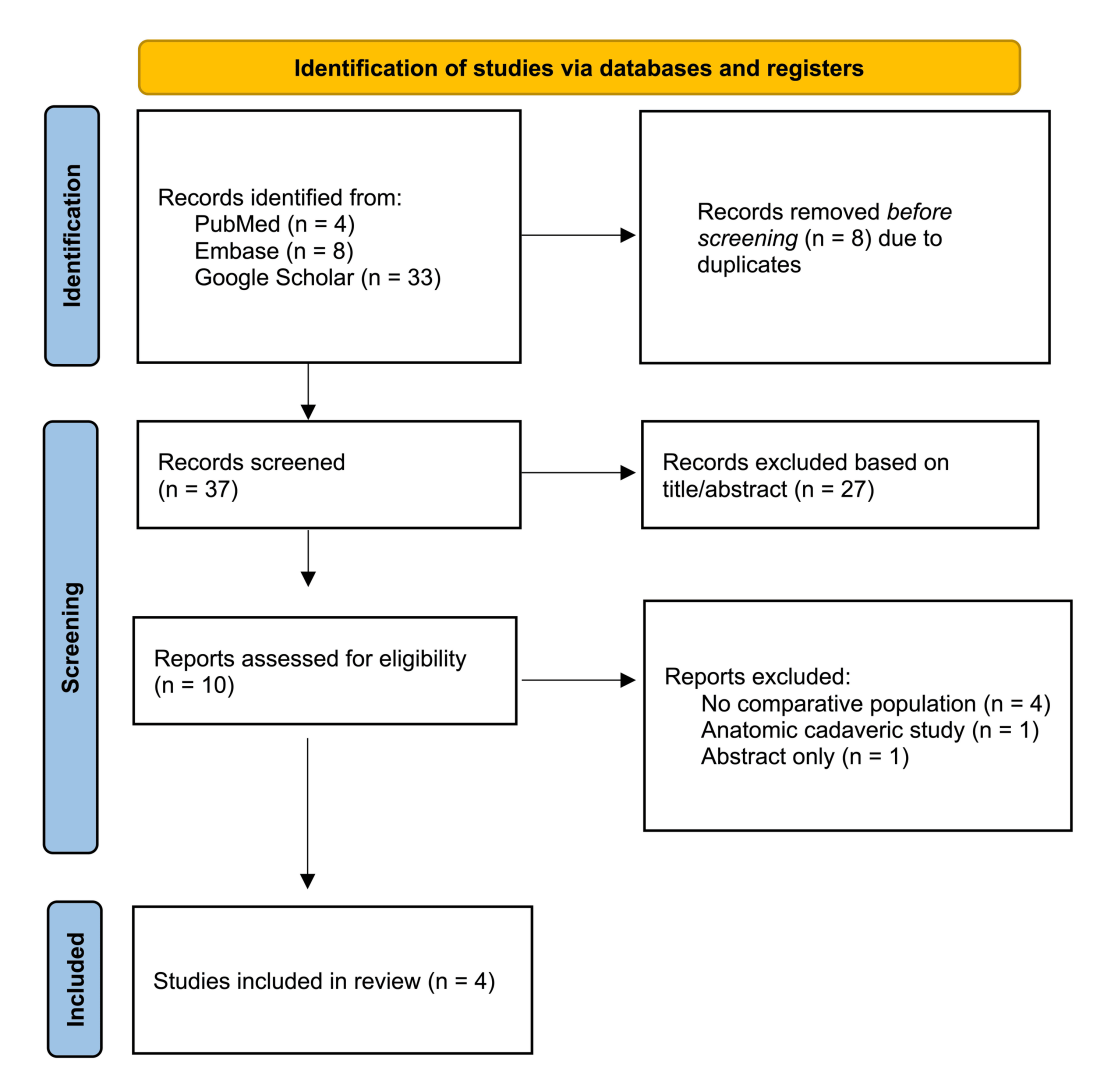
PRISMA flow diagram of study screening and selection PRISMA: Preferred Reporting Items for Systematic Review and Meta-analysis [17]

The systematic review followed the Preferred Reporting Items for Systematic Review and Meta-analysis (PRISMA) guidelines (Figure 1) [17] and the population, intervention, comparison, outcome (PICO) framework (Table 1). We included studies that analyzed HNS implantation via a two-incision versus a three-incision technique and reported surgical and device efficacy outcomes among adult patients diagnosed with moderate to severe OSA. Comparative experimental trials and retrospective cohorts were eligible; case reports and anatomic studies were excluded. Further, the studies that did not report measurable surgical or device efficacy outcomes were also excluded.

**Table 1:** The PICO framework PICO: Population, intervention, comparison, outcome; OSA: Obstructive sleep apnea; HNS: Hypoglossal nerve stimulator

Framework item	Study details
Patient population	Adult patients diagnosed with moderate to severe OSA
Intervention	HNS implantation via the two-incision technique
Comparison or control	HNS implantation via the three-incision technique
Outcome	The primary outcome of device efficacy included therapy usage, Apnea-Hypopnea Index,​ the Epworth Sleepiness Scale, and the Respiratory Sensing Lead Curve sleep waveform quality. The secondary surgical outcomes included the rates of follow-up revision surgery, operative time, and post-operative complications between the two techniques.

Study Outcomes

The primary clinical outcomes related to device efficacy included therapy usage, AHI,​ the Epworth Sleepiness Scale (ESS), and the Respiratory Sensing Lead Curve (RSC) sleep waveform quality [18,19]​. Therapy usage was measured as the average number of hours per night the patient used the HNS device [18,20]. Patients were provided with a handheld remote to activate the device each night before sleep and deactivate it upon waking, allowing for consistent control and usage monitoring ​[20].​ The AHI measures the average number of respiratory events per hour of sleep ​to compare sleep quality between the preoperative and postimplantation sleep studies conducted two to six months after surgery [18]. Lower AHI values indicate improved sleep outcomes. The ESS is a 24-point self-reported patient questionnaire assessing daytime sleepiness ​[18].​ Lower ESS scores reflect reduced sleepiness and greater therapeutic benefit. Sleep waveform quality measured via RSC evaluates the synchronization between HNS activation and the patient’s respiratory cycle, with greater alignment indicating better device responsiveness and sleep quality ​[19].​ 

The surgical outcomes included rates of follow-up revision surgery, operative time, and postoperative complications. The operative time was inconsistently defined across the studies as the duration between the initial incision and the beginning of wound closure, the time recorded from 'procedure start' to 'procedure finish,' or the interval from incision to postoperative application of sterile dressing. The variability in operative time limited the comparisons of surgery duration among the included studies. The postoperative complications were identified as events such as bleeding, infection, pneumothorax, and patient-reported issues (e.g., incisional pain, discomfort, and activity restriction). 

Search Strategy, Selection, and Data Extraction Process 

On November 21, 2024, we conducted a comprehensive literature search utilizing the electronic databases of PubMed, Embase, and Google Scholar. The study used the search terms and inclusion criteria outlined in Table 2 to ensure a broad and sensitive search. We reviewed the final list of studies to remove any duplicates. Two authors (AK and SM) independently evaluated titles and abstracts based on the inclusion and exclusion criteria, followed by a thorough review of the full-text articles. All authors assessed each full-text article for eligibility and resolved any disagreements through discussion or with input from the third reviewer (AS) as needed. The fourth author (RG) is a statistician and guided the design, methodology, data review, and interpretation. We used a Microsoft Excel (Microsoft Corp., Redmond, WA, USA) template to extract data such as author names and year, study design, sample size, patient demographics, and study outcomes from the final selected articles. Automation tools or artificial intelligence were not used to collect or manage data for this study. 

**Table 2:** Search terms and inclusion and exclusion criteria

Search terms	Publication date	Inclusion criteria	Exclusion criteria
PubMed: (("hypoglossal nerve stimulator implantation"[Title/Abstract] OR "hypoglossal nerve"[Title/Abstract]) AND ("obstructive sleep apnea"[Title/Abstract] OR OSA[Title/Abstract]) AND ("two incision"[Title/Abstract] OR "2-incision"[Title/Abstract]) AND ("three incision"[Title/Abstract] OR "3-incision"[Title/Abstract])) Embase: ((hypoglossal nerve stimulator implantation OR hypoglossal nerve) AND (obstructive sleep apnea OR OSA) AND ("two incision" OR "2-incision") AND ("three incision" OR "3-incision")) Google Scholar: allintitle OR allintext: ("hypoglossal nerve" OR "hypoglossal nerve stimulator implantation") ("obstructive sleep apnea" OR OSA) ("two incision" OR "2-incision") ("three incision" OR "3-incision")	No date restrictions	(1) Reporting direct comparisons between the two-incision vs. the three-incision technique; (2) Experimental clinical trials and observational retrospective studies	Case reports, case studies, and anatomic cadaveric studies

Data collection prioritized common outcomes across different studies to ensure consistency and comparability. The most comprehensive and clinically relevant data were extracted if multiple time points or analysis methods were available for an outcome. In case of inconsistencies between the studies, we selected the outcomes based on relevance to the research question. To guide the qualitative synthesis, the study outcomes were grouped into two categories: surgical and device outcomes. Additional variables of interest included demographic and clinical characteristics such as patient age, gender, and body mass index. We also extracted intervention details such as device model, stimulation parameters, and any modifications to the implantation technique. For missing data on device activation, we assumed the device was used according to established clinical guidelines, which recommend device activation four weeks post-implantation ​[21].​

Given the heterogeneity in outcome definitions and reporting, we conducted a prespecified qualitative review and analysis of the included studies. Where available, effects are presented as means with standard deviation or 95% confidence interval as reported by original authors. Meta‑analysis was not performed due to methodological and clinical heterogeneity and small cohort sizes. Subgroup (e.g., BMI, age, device model, learning‑curve epoch) and sensitivity analyses were prespecified but infeasible due to sparse, non‑harmonized reporting of results.

*Risk of Bias* 

For all the included studies, the risk of bias was assessed by two reviewers with adjudication using the Risk Of Bias In Non-Randomized Studies of Interventions (ROBINS-I) tool​ across seven domains [22]. The domains included confounding, selection of participants, classification of interventions, deviations from intended interventions, missing data, measurement of outcomes, and selection of the reported result. 

Results 

Search Results and Study Characteristics 

Four studies met the inclusion and exclusion criteria (Figure 1). All studies used the Inspire® upper airway stimulation device (Inspire Medical Systems Inc., Golden Valley, MN, USA) and employed a retrospective cohort study design. Table 3 presents an overview of the study characteristics, including study design, data source, sample size, patient demographics, and preoperative sleep quality measures.

**Table 3:** Characteristics of included studies ADHERE: Acute Decompensated Heart Failure National Registry, AHI: Apnea hypopnea index (events/hour)

Study	Study design	Data source	Incision group	Sample size (n)	Age (mean ± SD)	Male, n (%)	Female, n (%)	BMI (mean ± SD)	Preoperative AHI (mean ± SD)
Kent et al., 2021 [18]	Propensity score-matched retrospective cohort	Acute Decompensated Heart Failure National Registry (ADHERE) public data set	Three-incision	76	60.2 ± 9.8	53 (69.7%)	23 (30.3%)	29.3 ± 4.0	34.9 ± 14.5
			Two-incision	76	60.5 ± 12.0	47 (61.8%)	29 (38.2%)	29.2 ± 3.6	35.1 ± 14.6
Sagalow et al., 2022 [23]	Retrospective cohort	Single academic institution from November 2014 to June 2021	Three-incision	276	61.7 ± 11.2	188 (68.1%)	88 (31.9%)	28.8 ± 3.7	Not reported
			Two-incision	72	60.0 ± 13.3	50 (69.4%)	22 (30.6%)	28.3 ± 3.5	Not reported
Saltagi et al., 2022 [19]	Retrospective cohort	Single academic institution from June 2019 to September 2021	Three-incision	50	62.6 ± 11.2	26 (52%)	24 (48.0%)	29.4 ± 6.3	32.7 ± 13.9
			Two-incision	50	60.1 ± 13.0	29 (58%)	21 (42.0%)	29.3 ± 3.6	38.3 ± 18.8
Thakur et al., 2024 [24]	Retrospective cohort	Single academic institution from October 2020 to September 2022	Three-incision	5	55.2 ± 7.4	5 (100%)	0 (0.0%)	29.1 ± 2.2	39.0 ± 13.4
			Two-incision	16	59.3 ± 13.9	11 (69%)	5 (31.0%)	29.2 ± 2.3	41.9 ± 11.9

Surgical Outcomes

Table 4 presents a detailed breakdown of specific study outcome measures. The comparative results between the two- and three-incision techniques varied by specific outcome (Table 4). Two studies (Kent et al. and Sagalow et al.) reported statistically significant reductions in operative time with the two-incision approach ​[18,23].​ Kent et al. found that the average operative time decreased from 128.7 (95% CI: 124.5-132.9) to 86.6 (83.7-97.6) minutes (p < 0.001). Sagalow et al. reported a mean reduction in operative time from 143.3 ± 24.6 to 129.4 ± 24.3 minutes (p < 0.001). After adjusting for learning-curve effects using linear regression, the mean reduction was 13.9 minutes with the two-incision method compared to the three-incision method. Thakur et al. observed a decrease in operative time from 144 (95% CI: 112-176) to 134 (112-155) minutes, though this difference was not statistically significant (p = 0.276) ​[24].​ 

**Table 4:** Surgical outcome measures reported by included studies *A p-value <0.05 is considered statistically significant

Study	Operative time definition	Three-incision operative time, mean (95% CI or ± SD)	Two-incision operative time, mean (95% CI or ± SD)	Three-incision revision surgery n (%)	Two-incision revision surgery n (%)	Three-incision postop complications n (%)	Two-incision postop complications n (%)
Kent et al., 2021 [18]	Time from the first skin incision until the beginning of wound closure	128.7 (124.5-132.9)	86.6 (83.7-97.6)*	Not reported	Not reported	Minor: 10 (2.5%), Major: 4 (1.0%)	Minor: 4 (1.8%), Major: 2 (0.9%)
Sagalow et al., 2022 [23]	Time from 'procedure start' to 'procedure finish' using the electronic medical record (EMR) timestamps	143.3 ± 24.6	129.4 ± 24.3*	15.0 (5.4%)	0.0*	Patient-reported pain, discomfort, or activity restriction: 25 (9.1%)	Patient-reported pain, discomfort, or activity restriction: 4 (5.6%)
						Other discomfort: 18 (6.5%)	Other discomfort: 4 (5.6%)
Thakur et al., 2025 [24]	Incision to postoperative application of sterile dressing	144.0 (112.0-176.0)	134.0 (112.0-155.0)	Not reported	Not reported	None observed	None observed
Saltagi et el., 2023 [19]	Not reported	Not reported	Not reported	Not reported	Not reported	Not reported	Not reported

Device Efficacy Outcomes

Only one study (Kent et al.) reported AHI and ESS results and showed postoperative improvement in sleep quality in both techniques without statistical significance (p>0.05) [18]. The AHI decreased from 18.6 (95% CI: 15.1-22.1) to 10.6 (8.4-13.5) events/hour in the two-incision group and from 20.1 (95% CI: 16.2-24.0) to 9.3 (7.4-11.7) events/hour in the three-incision group. The ESS improvements were noted in both groups (mean decrease in ESS in two-incision: 2.7 (95% CI: 1.2-4.2) and three-incision: 4.3 (2.6-5.9)), with no statistical significance (p>0.05).

The statistically significant device outcomes included longer duration of device use by patients in the two-incision group (7.1 (95% CI: 6.6-7.6) vs. 6.2 (5.7-6.6) hours; p <0.01) [18]. Sagalow et al. identified a significantly lower revision surgery rate in the two-incision group (0%) than the three-incision group (5.4%, p = 0.048) [23]. Other significant results included waveform syncing with RSC to measure device function and efficacy in improving sleep quality by two studies [​19,24].​ Saltagi et al. reported statistically significantly improved waveform syncing and overall impression scores for sleep quality in the two-incision group (44% of patients) as compared to the three-incision group (34% of patients). Thakur et al. conducted a blinded evaluation of RSCs to compare waveform quality between the two techniques. Of the 16 patients in the two-incision group, 14 curves were rated as excellent and two as good. In the three-incision group, three out of five curves were rated excellent, and two were rated good. Notably, no curves in either group received a poor rating. However, the small sample size (n = 21) may limit the generalizability of this study’s findings [24]. 

Three studies reported post-op complications without statistical significance (Table 4). Kent et al. found low rates of minor and major complications in the two groups, which were not statistically significant ​[18].​ Total minor and major complications were slightly higher in the three-incision group (3.5%) than in the two-incision group (2.7%). Sagalow et al. found low, insignificant rates of infection, pneumothorax, and incisional pain between the two cohorts ​[23].​ A lower proportion of patients in the two-incision group (5.6%) reported pain, discomfort, or activity restriction than the three-incision group (9.1%). Thakur et al. observed no postoperative complications such as pneumothorax, infection, or discomfort in either group ​[24].

*Risk of Bias* 

Table 5 demonstrates the risk of bias using the ROBINS-I tool for all the included studies ​[22]. The risk of bias ranged from low to moderate across domains, with confounding concerns in studies with unbalanced covariates or small samples. One study (Sagalow et al.) exhibited a moderate risk of bias for confounding due to a lack of a baseline measure of sleep quality that could confound the measurement of change to postoperative improvement in sleep quality [​23].​ Another study (Thakur et al.) exhibited a high risk of bias due to confounding because of low sample size and unbalanced gender distribution between the two groups ​[24].​ The remaining six domains of risk of bias were rated low. Although the two‑incision technique was introduced later, it does not eliminate the possibility of selection bias. Differences in surgeon experience, institutional adoption pathways, and temporal learning‑curve effects may have influenced which patients received each technique. One study was propensity score matched, and other studies found no significant differences in the baseline demographic and sleep quality between the two groups. Also, both surgeries were conducted as per standardized protocols, and the outcomes were measured in a standardized manner. All outcomes stated in the objectives were reported in the results, with no missing data in all of the included studies. 

**Table 5:** ROBINS-I risk of bias ratings across seven domains for the included studies ROBINS-I: Risk Of Bias In Non-Randomized Studies of Interventions

Study	Confounding	Selection bias	Intervention classification bias	Deviations from intended intervention	Missing data	Outcome measurement bias	Selective reporting of results
Kent et al., 2021 [18]	Low	Low	Low	Low	Low	Low	Low
Sagalow et al., 2022 [22]	Moderate	Low	Low	Low	Low	Low	Low
Saltagi et al., 2022 [19]	Low	Low	Low	Low	Low	Low	Low
Thakur et al., 2024 [24]	High	Low	Low	Low	Low	Low	Low

Discussion

Our systematic review, based on limited retrospective studies, suggests that the two-incision technique for HNS implantation represents a potential alternative to the traditional three-incision approach in selected clinical settings. Findings from three retrospective cohorts indicate that a two‑incision HNS implantation can reduce operative time without compromising short‑term safety or device performance relative to the traditional three‑incision technique [18,23,24]. Reduction in operative time may reduce anesthesia exposure and surgical trauma, especially in populations with an increased risk of perioperative complications, with potential implications for operative efficiency and health-system resource utilization [2,3]. Training appears incremental, focusing on consolidated chest‑wall sensing‑lead placement and avoidance of malposition. Formal cost‑effectiveness analyses and implementation studies are needed to quantify these effects.

Limited evidence from two studies on improved waveform syncing suggests that the two-incision technique may offer comparable device performance, although these findings are based on small samples and should be interpreted cautiously. Our study results suggest that the nerve stimulator sensing electrode can be implanted via a two-incision technique without interference with respiratory signals [19,24]. Additionally, some evidence suggests that the two-incision technique may lead to fewer corrective surgeries, suggesting potential advantages related to durability and reduced long-term surgical complications [23]. 

We found that patient-reported sleep outcomes improved in both techniques but did not demonstrate statistically significant differences. Both approaches utilize identical functional components (stimulation lead on the hypoglossal nerve, thoracic sensing lead, and pulse generator), with the principal difference being the number of incisions and lead routing. While similar postoperative AHI and ESS results are biologically plausible, equivalence between techniques cannot be established based on the current evidence. Accordingly, given low-to-moderate certainty (observational cohorts, small samples, heterogeneous reporting), two-incision HNS may be considered where appropriate expertise exists, with broader adoption contingent upon confirmatory prospective, adequately powered, and economic studies.

This systematic review has several important limitations. First, the evidence base is limited to four retrospective cohort studies, with no randomized controlled trials, making the findings susceptible to residual confounding. In particular, surgeon experience and procedural learning-curve effects may have influenced operative time, complication rates, and revision surgery outcomes, as the two-incision technique was introduced later and may have been preferentially adopted by more experienced surgeons [22]. Second, all included studies evaluated a single HNS device (Inspire®), which may limit generalizability to other current or future HNS systems with differing device designs or implantation workflows [24]. Third, none of the included studies assessed cost-effectiveness or broader health-system impacts, such as resource utilization or reimbursement considerations. While reduced operative time may suggest potential economic advantages, these effects cannot be inferred without formal economic analyses. Additional limitations include heterogeneous operative time definitions and small sample sizes in some cohorts and the absence of subgroup or sensitivity analyses to evaluate potential effect modifiers. 

Future research should aim to address these limitations, as there is increasing evidence on the efficacy of this novel technique [15,19,23,24]. Our study findings apply to US patients and do not fully reflect the global disease burden and application. Despite these limitations, this review provides, to the best of our knowledge, the first focused synthesis comparing two-incision and three-incision techniques for HNS implantation in OSA. 

## Conclusions

Based on limited retrospective evidence, the two-incision technique required a shorter operative time, without evidence of worsened short-term sleep outcomes compared with the three-incision approach. Additionally, the two-incision group tended to use the device for longer durations each night, though the clinical significance of this finding remains uncertain. The two-incision technique was also associated with a lower reported revision surgery rate; minor and major postoperative complications were also low but not significant. Improvements in sleep quality measures, including AHI and ESS, were modest and did not reach statistical significance. Given the limited retrospective evidence and absence of randomized trials, these findings should be interpreted as preliminary. Furthermore, the current evidence base is limited by small sample sizes and heterogeneous outcome definitions. Future prospective, randomized, and adequately powered studies are needed to confirm clinical equivalence, assess long-term durability, and evaluate patient-centered outcomes between the two surgical techniques.
